# Bronchiolitis Admissions in a Lebanese Tertiary Medical Center: A 10 Years' Experience

**DOI:** 10.3389/fped.2019.00189

**Published:** 2019-05-17

**Authors:** Zeina Naja, Danielle Fayad, Sarah Khafaja, Sarah Chamseddine, Ghassan Dbaibo, Rima Hanna-Wakim

**Affiliations:** ^1^Department of Pediatrics and Adolescent Medicine, American University of Beirut Medical Center, Beirut, Lebanon; ^2^Division of Pediatric Infectious Diseases, Department of Pediatrics and Adolescent Medicine, American University of Beirut Medical Center, Beirut, Lebanon; ^3^Center for Infectious Diseases Research, American University of Beirut, Beirut, Lebanon

**Keywords:** bronchiolitis, respiratory syncytial virus, risk factors, hospitalization, retrospective

## Abstract

Bronchiolitis and more specifically respiratory syncytial virus (RSV) bronchiolitis is a leading cause of global childhood morbidity and mortality. Despite the previous identification of possible risk factors associated with the severity of bronchiolitis, the data from Lebanon remains limited. We described the burden of bronchiolitis hospitalizations in children under 5 years of age in a tertiary care center in Lebanon from October 2004 to October 2014 and identified the risk factors associated with severe bronchiolitis. This was a retrospective cohort study conducted at the American University of Beirut Medical Center. Records of children younger than 5 years of age admitted with a diagnosis of bronchiolitis were reviewed. More than half the patients were RSV positive. RSV bronchiolitis was found to be significantly associated with longer hospital stay compared to children with non-RSV bronchiolitis (*P* = 0.007). Children exposed to smoking had an increased risk for longer hospital stay (*P* = 0.002) and were more likely to require ICU admission (*P* < 0.001) and supplemental oxygen (*P* = 0.045). Congenital heart disease was found to be a significant risk factor for severe bronchiolitis (*P* < 0.005).

**Conclusion:** Patients with RSV bronchiolitis had a longer hospital stay compared to patients with non-RSV bronchiolitis. Exposure to smoking was associated with a more severe and complicated RSV infection. Congenital heart disease was the only risk factor significantly associated with all markers of bronchiolitis disease severity.

## Introduction

Lower respiratory tract infection (LRTI), including bronchiolitis and pneumonia, constitute the leading cause of global child morbidity and mortality (1). Bronchiolitis is considered the most common respiratory disease in young children and is a major cause of hospitalization (2). From 1997 to 2006 in the United States, the average annual rate of bronchiolitis among children younger than 5 years of age was 27.9 per 1,000 with half a million annual hospitalizations (3). Hospital costs for care related to bronchiolitis in children younger than 5 years of age have been on the rise in the United States and exceeded 1.7 billion US dollars in 2009 (2).

Respiratory Syncytial Virus (RSV) is the most common pathogen causing bronchiolitis in young children and the most frequent cause of hospitalization within the pediatric population in developed countries (4). An estimated 33.8 million new episodes of RSV infections occur in children under 5 years of age worldwide accounting for 22% of LRTI episodes. Of those, 3.4 million episodes represent severe disease and require hospital admission (5). Moreover, according to a prospective population-based surveillance study of acute respiratory infections in children <5 years conducted in three U.S. counties, RSV was associated with 20% of hospitalizations, 18% of emergency department visits, and 15% of office visits for acute respiratory infections (6).

Contrary to non-RSV respiratory disease, RSV infection frequently leads to serious complications. Two retrospective studies conducted in two tertiary medical centers in Texas in 2010 and in Spain in 2012 over periods of 5 and 10 years, respectively, compared the rate of hospitalization and outcomes of RSV and non-RSV bronchiolitis in children under the age of 2 years (1, 7). Both studies concluded that RSV bronchiolitis was more severe than non-RSV bronchiolitis in terms of need for supplemental oxygen, intensive care unit (ICU) admission and length of hospital stay (1, 7).

Across the Middle East and North Africa (MENA) region, RSV imposes a significant burden of disease as well, and has been commonly identified in hospitalized infants and children with bronchiolitis (8). In a study involving 3,168 Jordanian pediatric patients, RSV was positive in 44% (9). High prevalence rates of hospitalizations due to RSV have been reported in Turkey, Egypt, and Lebanon as well (10–13). The severity of RSV bronchiolitis varies between studies, and data highlighting the risk factors for severity is still lacking.

To date, a variety of demographic features and risk factors have been associated with a higher likelihood of RSV bronchiolitis (7). Recent studies are exploring chronic conditions as possible risk factors associated with increased risk of severe RSV infection (14).

The aim of this study is to provide a comprehensive view of the epidemiologic characteristics and burden of bronchiolitis in hospitalized children younger than 5 years of age admitted to a tertiary care center in Beirut-Lebanon, and to examine the risk factors, clinical features, outcomes, and severity of RSV vs. non-RSV bronchiolitis.

## Materials and Methods

### Study Design

This was a retrospective cohort study conducted at the American University of Beirut Medical Center (AUBMC) located in Beirut, Lebanon. The study was approved by the institutional review board (IRB) at AUBMC (IRB approval number was PED.GD.08).

AUBMC is a tertiary medical care center located in Beirut, the capital of Lebanon.

It has a total of 420 hospital beds, including 28 pediatric inpatients beds, 21 neonatal intensive care unit (NICU) beds, 10 pediatric (PICU) beds; in addition there are 18 hospital beds in the inpatient cancer unit. AUMBC accommodates around 9,500 pediatric inpatient admissions to these different units per year, that present from different areas all around Lebanon.

Most of the hospital admissions during the study period were covered by private medical insurances (around 45%), and the rest by public governmental coverage or by self-payers.

All patients were identified retrospectively and their charts reviewed manually through medical records by looking at the following ICD-9 codes for discharge diagnosis: “Bronchiolitis,” “RSV bronchiolitis,” or “RSV infection.” Children <5 years of age with one of the above discharge diagnosis, admitted to the hospital between October 1st, 2004 and October 31st, 2014, were included in the study.

### Data Collection

Data collected included the following information: basic demographic and epidemiologic characteristics (age, gender, year, and month of hospital admission, breastfeeding, exposure to secondhand smoking, previous NICU admission, previous history of intubation), presence of underlying medical conditions [including prematurity, chronic lung disease of prematurity, congenital heart disease (CHD), primary or secondary immunodeficiencies, cystic fibrosis, neuromuscular disorders, and asthma], hospital stay (regular floor/ICU, length of hospital/ICU stay). In addition, data was collected on outcomes including requirement and duration of supplemental oxygen [by nasal cannula, facemask, high-flow nasal cannula, nasal continuous positive airway pressure ventilation (CPAP), intubation, and mechanical ventilation], need and length of mechanical ventilation, and mortality.

In order to investigate dissimilarities in disease severity between RSV and non-RSV bronchiolitis, we explored multiple parameters including length of hospital stay in days, need for supplemental oxygen including oxygen by nasal cannula, facemask, high-flow nasal cannula, nasal CPAP, intubation, and mechanical ventilation, need and length of ICU admission as well as need and duration of mechanical ventilation.

The laboratory diagnosis of RSV was made on respiratory secretions by the use of rapid assay utilizing antigen capture technology (SAS^TM^ RSV Alert test by SA Scientific, San Antonio, Texas, USA). The same test was used during the whole study period. The sensitivity and specificity of the test are 83.5 and 91.3%, respectively (15).

### Statistical Analysis

Descriptive analyses were performed by using frequency distributions or rates. Means [± standard deviation (SD)] were used to summarize the demographic data and patients' baseline characteristics.

Statistical analysis was performed using Pearson Chi-square test and the Student's *t*-test to compare the demographic and epidemiologic characteristics between hospitalized children with RSV and non-RSV bronchiolitis. For variables with count <5 cases, Chi-square Fisher's exact test was used. Predictors of disease severity regardless of the etiology of bronchiolitis were then studied using binary logistic regressions. All covariates that showed a *p*-value <0.30 at the bivariate level were added in the final logistic regression model. Disease severity was defined as showing separately one of the four following outcomes: longer stay at the hospital above 7 days, need for oxygen supplementation, need for ICU admission and need for ventilatory support.

A *p*-value <0.05 was considered a statistically significant result. All statistical analyses were performed with the use of the Statistical Package for Social Sciences (SPSS) program, version 23.0 for Windows (IBM, Armonk, NY).

## Results

### Study Group

**Figure 1:**
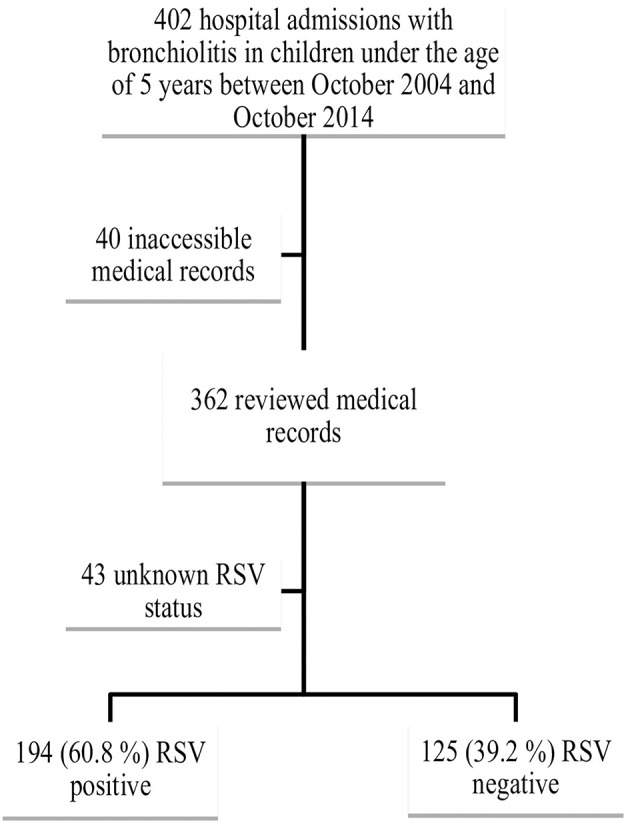
Identification and inclusion of cases in the study. RSV, respiratory syncytial virus.

We identified 402 cases of children below the age of 5 years with an admission diagnosis of “bronchiolitis,” “RSV bronchiolitis,” or “RSV infection” admitted to the hospital during the study period. The medical records of 362 patients were available for review; 43 patients were excluded from the final analysis because of unknown RSV status leaving a final study cohort of 319 patients for statistical analysis. Among the 319 patients, 194 were RSV positive (60.8%) and 125 were RSV negative (39.2%) (Figure 1).

To note that out of 402 patients, 11 patients were readmitted for bronchiolitis during different seasons.

### Seasonal Distribution

**Figure 2:**
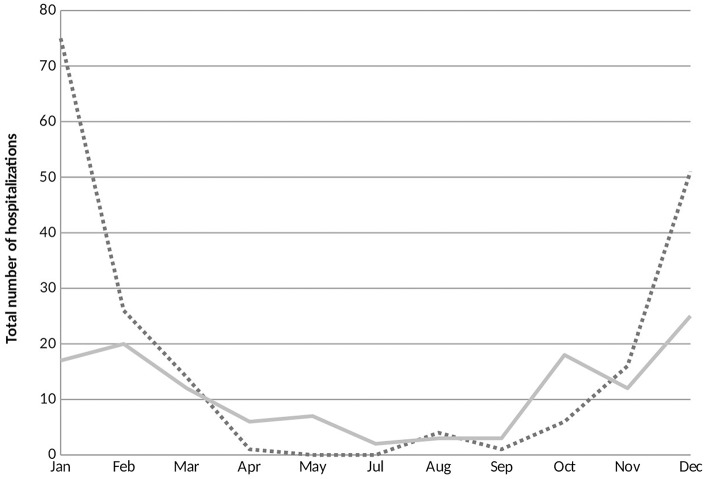
Seasonal variations of respiratory syncytial virus (RSV) and non-RSV associated bronchiolitis hospitalizations. The horizontal axis represents the months of the year and the vertical axis exhibits the total number of RSV and non-RSV bronchiolitis cases hospitalized during the study period. RSV, respiratory syncytial virus. ….RSV positive; —— RSV negative.

RSV hospitalizations started to rise at the end of October and early November and peaked during the months of January and February. A small number of RSV bronchiolitis cases were diagnosed and hospitalized off season along the year. Contrariwise, cases of non-RSV bronchiolitis hospitalizations were most frequent during the month of December and displayed a dispersed distribution throughout the year with no significant peak in hospital admissions (Figure 2).

### Demographic Characteristics

The demographic characteristics and features of the patients with RSV and non-RSV bronchiolitis are summarized in Table 1.

**Table 1:** Demographic characteristics and risk factors of children younger than 5 years of age hospitalized with bronchiolitis (RSV and non-RSV) during the study period.

	**RSV bronchiolitis (*N* = 194)**	**Non-RSV bronchiolitis (*N* = 125)**	***p*-value*^†^***
**DEMOGRAPHIC CHARACTERISTICS**			
Mean age in months (*SD*)	7.05 (9.06)	7.01 (6.42)	0.958
**Gender**, ***n*** **(%)**
Male	109 (56.2)	64 (51.2)	0.383
Female	85 (43.8)	61 (48.8)	
**RISK FACTORS**, ***N*** **(%)**			
Prematurity, ***N*****'** = 317	37 (19.2)	23 (18.5)	0.890
Breastfeeding^a^, ***N*****'** = 261	80 (52.6)	41 (37.6)	**0.016**
Exposure to smoking^b^, ***N*****'** = 222	32 (25.4)	24 (25.0)	0.946
Previous NICU admission, ***N*****'** = 308	32 (17.4)	28 (22.6)	0.259
Previous history of intubation, ***N*****'** = 297	8 (4.4)	7 (6.0)	0.554
Congenital heart disease, ***N*****'** = 318	14 (7.3)	8 (6.4)	0.769
Chronic lung disease of prematurity, ***N*****'** = 318	3 (1.6)	5 (4.0)	0.271^*^
Asthma, ***N*****'** = 318	6 (3.1)	0 (0)	0.085

*Significant differences for p-values are indicated in bold*.

*N' represents information available on the variable*.

† *Pearson Chi-Square was used (no expected count <5)*.

* *Fisher's exact test was used (at least one expected count <5)*.

a *Breastfeeding: data collected on 261 patients (152 with RSV bronchiolotis and 109 with non-RSV bronchiolitis)*.

b *Exposure to smoking: data collected on 222 patients (126 with RSV bronchiolitis and 96 with non-RSV bronchiolitis)*.

*RSV, respiratory syncytial virus; NICU, neonatal intensive care unit*.

The mean age at diagnosis of bronchiolitis was similar between the two groups (7.05 ± 9.06 and 7.01 ± 6.42 months [mean, ± SD]) in patients with RSV and non-RSV bronchiolitis, respectively. There were no differences in the percentage of premature patients between the two groups (19.2 and 18.5%, respectively, *P* = 0.89). More males were diagnosed with RSV bronchiolitis but this difference did not reach statistical significance.

### Risk Factors in Children Hospitalized With Bronchiolitis

The risk factors reported in the literature known to be associated with bronchiolitis were compared in patients hospitalized with RSV and non-RSV bronchiolitis (Table 1).

Breastfeeding was more frequent among patients with RSV bronchiolitis compared to patients with non-RSV bronchiolitis (52.6 vs. 37.6%, *P* = 0.02), but passive smoking was not found to be a statistically significant risk factor for RSV bronchiolitis.

Chronic lung disease of prematurity was more frequent in patients with non-RSV bronchiolitis compared to RSV bronchiolitis (4 vs. 1.6%). Nevertheless, this difference did not reach statistical significance (*P* = 0.27). All asthmatic patients included in the study tested positive for RSV but asthma was not found to be a statistically significant risk factor for RSV bronchiolitis (*P* = 0.08). Other suggested risk factors including congenital heart disease and malignancy were comparable between the two groups.

### Management Modalities

Management modalities were compared in patients hospitalized with RSV and non-RSV bronchiolitis (Table 2).

**Table 2:** Management modalities used in children younger than 5 years of age hospitalized with RSV and non-RSV bronchiolitis from 2004 to 2014.

**Management modalities**	**RSV bronchiolitis, n (%) (*N* = 194)**	**Non-RSV bronchiolitis n(%) (*N* = 125)**	***p*-value**
Inhaled beta-agonist	155 (79.9)	103 (82.4)	0.58
Inhaled steroids	18 (9.3)	12 (9.6)	0.92
Systemic steroids	33 (17.0)	41 (32.8)	**0.001**
Antibiotics	108 (55.7)	55 (44.0)	**0.042**

*Significant differences for p-values are indicated in bold*.

*Pearson Chi-Square was used (no expected count <5)*.

*RSV, respiratory syncytial virus*.

Inhaled bronchodilators were used in 80.8% of the patients (79.9 and 82.4%, *P* = 0.58 in RSV and non-RSV bronchiolitis, respectively). Systemic steroids were more commonly used in non-RSV bronchiolitis patients compared to RSV bronchiolitis patients (*P* = 0.001). Antibiotics were used in half of the patients hospitalized with RSV bronchiolitis and non-RSV bronchiolitis. The most commonly used antibiotics were Ceftriaxone, Ampicillin and Clarithromycin. The mean duration of antibiotic use was 4.64 days.

### Disease Severity in RSV and Non-RSV Bronchiolitis

RSV bronchiolitis was found to be associated with a longer hospital stay (*P* < 0.05). Moreover, children diagnosed with RSV bronchiolitis spent a longer period in ICU compared to children with non-RSV bronchiolitis but this difference did not reach statistical significance (*P* = 0.18). Need for supplemental oxygen and ventilatory support were comparable between the two groups (Table 3).

**Table 3:** Disease severity characteristics in children younger than 5 years of age hospitalized with bronchiolitis (RSV and non-RSV) from 2004 to 2014.

**Parameters of disease severity**	**RSV bronchiolitis (*N* = 194)**	**Non-RSV bronchiolitis (*N* = 125)**	***p*-value**
Length of hospital stay, mean (SD), days	7.48 (6.73)	5.60 (4.86)	**0.007**
Supplemental oxygen, *n* (%)	82 (42.3)	43 (34.4)	0.160
Nasal CPAP, *n* (%)	4 (2.1)	0 (0)	0.158
ICU admission, *n* (%)	39 (20.1)	19 (15.2)	0.268
ICU admission duration, mean (SD), days	8.97 (9.91)	5.68 (5.34)	0.182
Invasive ventilatory support need, *n* (%)	17 (8.8)	5 (4.0)	0.101
Mortality, *n* (%)	2 (1.0)	1 (0.8)	1.000

*Significant differences for p-values are indicated in bold*.

*RSV, respiratory syncytial virus; CPAP, continuous positive airway pressure; ICU, intensive care unit*.

### Risk Factors for Severe RSV Disease

RSV diagnosis was significantly associated with a longer hospital stay (*P* < 0.05). Children exposed to secondhand smoking had an increased risk for longer hospital stay (*P* < 0.05). They were also more likely to require ICU admission (*P* < 0.001) and supplemental oxygen (*P* < 0.05). CHD was associated with all markers of disease severity (Table 4).

**Table 4:** Odds ratios (ORs) for risk factors associated with disease severity in hospitalized children younger than 5 years of age hospitalized from 2004 to 2014.

**Predictors**	**Hospital stay longer than 7 days**		**Need of supplemental oxygen**		**Need of ICU admission**		**Need of ventilatory support**	
	**OR [95% CI]**	***p*-value**	**OR [95% CI]**	***p*-value**	**OR [95% CI]**	***p*-value**	**OR [95% CI]**	***p-*value**
RSV	2.50 [1.15–5.14]	**0.02**	1.22 [0.68–2.20]	0.506	1.29 [0.56–2.96]	0.547	1.11 [0.27–4.57]	0.888
**DEMOGRAPHIC CHARACTERISTICS**								
Age	1.00 [0.96–1.04]	0.921	1.03 [0.99–1.06]	0.181	1.02 [0.98–1.07]	0.286	1.04 [0.99–1.10]	0.133
Male	1.43 [0.70–2.95]	0.327	0.80 [0.45–1.45]	0.472	1.51 [0.67–3.44]	0.323	0.86 [0.21–3.56]	0.835
**RISK FACTORS**								
Prematurity	1.70 [0.70–4.22]	0.249	1.83 [0.87–3.88]	0.112	3.09 [1.19–8.07]	**0.021**	1.60 [0.29–8.83]	0.591
Congenital heart disease	7.31 [2.20–24.26]	**0.001**	11.15 [2.37–52.40]	**0.002**	15.33 [4.20–56.02]	**<0.001**	12.34 [2.68–56.74]	**0.001**
Exposure to smoking	3.36 [1.59–7.10]	**0.002**	2.20 [1.15–4.22]	**0.017**	5.32 [2.27–12.50]	**<0.001**	3.49 [0.85–14.29]	0.082

*Significant differences for p-values are indicated in bold*.

*OR, Odds Ratio; CI, Confidence Interval*.

## Discussion

Bronchiolitis is a leading cause of hospitalization in infants and young children (2). In our study, a total of 319 patients hospitalized at AUBMC during the study period from October 2004 till October 2014 with a diagnosis of bronchiolitis, RSV bronchiolitis or RSV infection were included in the final analysis. RSV bronchiolitis was diagnosed in 61% of the cohort. This value was higher than the one reported by Assaf-Casals et al. at another tertiary care center in Beirut (22.1%) between 2012 and 2014 (12).

In contrast with a previous study conducted in Lebanon (12), we found that the seasonality patterns for RSV and non-RSV bronchiolitis hospitalizations were somewhat different. Whereas Assaf-Casals et al. described peak prevalence for both subgroups during the month of December; RSV hospitalizations in our study peaked higher and later than non-RSV hospital admissions. These findings are in accordance with previous studies conducted in Israel (16), Turkey (17), and Jordan (9) which showed that RSV hospitalizations were most common during the first 3 months of the year.

A variety of demographic features and risk factors were associated with a higher likelihood of acquiring RSV bronchiolitis. Recently, in a US multicenter prospective cohort study of 1,836 pediatric patients with bronchiolitis, 60% of the patients were males and the mean age at diagnosis was 4 months (18). Our study also revealed male predominance which is expected since male infants are suspected to have decreased pulmonary function compared to female infants (19). However, we found that the rates of bronchiolitis requiring hospitalization were higher during infancy with an older mean age at admission of 7 months for both RSV and non-RSV subgroups. Although prematurity is evidently recognized as a predisposing factor for RSV bronchiolitis (2, 14, 20); our data did not show significantly higher rates of hospitalization for RSV and non-RSV bronchiolitis among preterm infants.

The role of breastfeeding in protecting against RSV bronchiolitis and preventing hospitalization and death has been very well-established (14, 20–22). Surprisingly, we found that patients with RSV bronchiolitis were more likely to be breastfed compared to patients with non-RSV bronchiolitis. This result might be lacking in accuracy due the retrospective nature of our study and possibility of improper charting. Moreover, information on duration of breastfeeding was unavailable for review and was not included in the analysis. In addition, we did not have accurate assessment of the prevalence of breastfeeding in a control group of patients without bronchiolitis. Another limitation of the above observation is that patients with non-RSV bronchiolitis did not have during the study period additional testing (like molecular assays to determine if they have RSV or other viruses). Regardless of these results and according to the 2014 American Academy of Pediatrics (AAP) guidelines for the management of bronchiolitis, all mothers should be encouraged to breastfeed for at least 6 months to prevent lower respiratory tract infections (23).

Although associations between exposure to smoking and lower respiratory tract infections in children have been documented in the literature, many investigators have not found an increase in the risk of acquiring RSV-bronchiolitis in children exposed to secondhand smoking (12, 24, 25) which is in accordance with our findings. Several studies showed that second-hand smoke exposure is associated with increased severity of RSV infection (26–32).

In agreement with previous studies (7, 12, 33), we found that RSV infections led to a longer hospital course. This finding underscores the need for a treatment modality against RSV infection in infants. The only humanized monoclonal antibody Palivizumab has been approved in 1998 by the US Food and Drug Administration and is being used for RSV infection prevention in children at high risk for the disease (34). The AAP issued an updated guidance on Palivizumab prophylaxis in high risk infants and young children in which it did not consider healthy preterm infants born at 29 and 35 weeks as candidates for Palivizumab prophylaxis (34). Ongoing research is currently focusing on characterizing pediatric subpopulations that should be offered immunoprophylaxis (35).

According to the most recent AAP guidelines for the diagnosis and management of bronchiolitis; bronchiolitis is a clinical diagnosis and does not require routine laboratory or radiologic tests for diagnosis (23). The routine use of multiple management modalities including bronchodilators, antibiotics and corticosteroids have not been shown to be effective and should be avoided (36). In our study, inhaled bronchodilators and antibiotics were frequently used in RSV and non-RSV bronchiolitis patients. However, it is difficult to assess whether the usage of those therapies was warranted due to the retrospective nature of our study. Regardless of our findings, physicians should abide by the evidence-based guidelines and not regularly prescribe bronchodilators, antibiotics, and/or corticosteroids to children with bronchiolitis (23).

In our study, infants exposed to secondhand cigarette smoking appeared to have a predilection to develop severe bronchiolitis after acquiring RSV infection. Similar results were highlighted in a study by Gurkan et al. in which infants admitted with serious RSV bronchiolitis were found to have higher serum cotinine levels compared to infants admitted for other causes (37). Along these lines, it is imperative for physicians and pediatricians to inform parents of the risks associated with secondhand smoking exposure and encourage them to avoid such a practice. In a population-based retrospective cohort study of 764 children younger than 2 years of age admitted with bronchiolitis, CHD was associated with a 50% longer hospital stay compared to patients without cardiac problems (38). In agreement with those observations, we found that CHD was associated with more severe bronchiolitis in terms of hospital stay, need for ICU admission, supplemental oxygen and ventilatory support.

### Limitations of the Study

The retrospective design of our study limited our ability to collect accurate and complete data on demographic characteristics and risk factors. This study enrolled patients from only one tertiary care center in Lebanon and not multiple ones.

In addition, we had low numbers of young infants who are mostly at risk of severe bronchiolitis. Moreover, the results on disease severity should be carefully interpreted since a diagnosis of RSV could have biased the management of these patients leading to a longer hospital stay, ICU admission and more invasive interventions. We had also limited numbers of patients during the study period who were managed with CPAP or high-flow nasal cannula. Furthermore, some of the non-RSV bronchiolitis cases might have had RSV since we were using RSV antigen testing for the diagnosis of the cases in our medical center and not Polymerase Chain Reaction (PCR) based diagnostic technologies.

## Conclusion

More than half of patients with bronchiolitis were RSV positive. Patients with RSV Bronchiolitis had a longer hospital stay compared to patients with non-RSV bronchiolitis. Exposure to smoking was associated with a more severe and complicated RSV infection. CHD was the only risk factor significantly associated with all markers of disease severity.

## Ethics Statement

The study was approved by the institutional review board (IRB) at the American University of Beirut Medical Center (IRB approval number was PED.GD.08).

No informed consent needed as this is a retrospective study.

## Author Contributions

ZN and RH-W conceived the presented idea and designed the study methods. ZN and SC took lead in data collection from medical records. DF contributed to reviewing the collected data and took the lead in manuscript writing. SK conducted and verified all statistical analyses via SPSS. RH-W and GD supervised the entire process and closely monitored progress of the study. All authors have read and approved the final manuscript.

### Conflict of Interest Statement

The authors declare that the research was conducted in the absence of any commercial or financial relationships that could be construed as a potential conflict of interest.

## Abbreviations

RSV: Respiratory Syncytial Virus
AUBMC: American University of Beirut
LRTI: Lower respiratory tract infection
ICU: Intensive care unit
AAP: American Academy of Pediatrics
CHD: Congenital heart disease.

## References

[1] Hervás D Reina J Yañez A del Valle JM Figuerola J Hervás JA. Epidemiology of hospitalization for acute bronchiolitis in children: differences between RSV and non-RSV bronchiolitis. Eur J Clin Microbiol Infect Dis. (2012) 31:1975–81. 10.1007/s10096-011-1529-y 22240853

[2] Hasegawa K Tsugawa Y Brown DF Mansbach JM Camargo CA. Trends in bronchiolitis hospitalizations in the United States, 2000-2009. Pediatrics. (2013) 132:28–36. 10.1542/peds.2012-3877 23733801 PMC3691534

[3] Stockman LJ Curns AT Anderson LJ Fischer-Langley G. Respiratory syncytial virus-associated hospitalizations among infants and young children in the United States, 1997-2006. Pediatr Infect Dis J. (2012) 31:5–9. 10.1097/INF.0b013e31822e68e6 21817948

[4] Shi T McAllister DA O'Brien KL Simoes EAF Madhi SA Gessner BD. Global, regional, and national disease burden estimates of acute lower respiratory infections due to respiratory syncytial virus in young children in 2015: a systematic review and modelling study. Lancet. (2017) 390:946–58. 10.1016/S0140-6736(17)30938-8 28689664 PMC5592248

[5] Nair H Nokes DJ Gessner BD Dherani M Madhi SA Singleton RJ. Global burden of acute lower respiratory infections due to respiratory syncytial virus in young children: a systematic review and meta-analysis. Lancet. (2010) 375:1545–55. 10.1016/S0140-6736(10)60206-1 20399493 PMC2864404

[6] Hall CB Weinberg GA Iwane MK Blumkin AK Edwards KM Staat MA. The burden of respiratory syncytial virus infection in young children. N Engl J Med. (2009) 360:588–98. 10.1056/NEJMoa0804877 19196675 PMC4829966

[7] García CG Bhore R Soriano-Fallas A Trost M Chason R Ramilo O. Risk factors in children hospitalized with RSV bronchiolitis versus non-RSV bronchiolitis. Pediatrics. (2010) 126:e1453–60. 10.1542/peds.2010-0507 21098154 PMC3761792

[8] Horton KC Dueger EL Kandeel A Abdallat M El-Kholy A Al-Awaidy S. Viral etiology, seasonality and severity of hospitalized patients with severe acute respiratory infections in the Eastern Mediterranean Region, 2007-2014. PLoS ONE. (2017) 12:e0180954. 10.1371/journal.pone.0180954 28704440 PMC5509236

[9] Halasa N Williams J Faouri S Shehabi A Vermund SH Wang L. Natural history and epidemiology of respiratory syncytial virus infection in the Middle East: hospital surveillance for children under age two in Jordan. Vaccine. (2015) 33:6479–87. 10.1016/j.vaccine.2015.08.048 26314623 PMC7115487

[10] Gokce S Kurugol Z Koturoglu G Cicek C Aslan A. Etiology, seasonality, and clinical features of viral respiratory tract infections in children hospitalized with acute bronchiolitis: a single-center study. Glob Pediatr Health. (2017) 4:2333794X17714378. 10.1177/2333794X17714378 28680946 PMC5484425

[11] Othman HT Abu Elhamed WA Hassan DM Soliman MS Abdel Baset RW. Respiratory syncytial virus and human metapneumovirus in severe lower respiratory tract infections in children under two. J Infect Dev Countries. (2016) 10:283–9. 10.3855/jidc.7087 27031461

[12] Assaf-Casala A Ghanem S Rajab M Respiratory syncytial virus: prevalence and features among hospitalized lebanese children. Br J Med Med Res. (2015) 6:77–8. 10.9734/BJMMR/2015/12608

[13] Finianos M Issa R Curran MD Afif C Rajab M Irani J. Etiology, seasonality, and clinical characterization of viral respiratory infections among hospitalized children in Beirut, Lebanon. J Med Virol. (2016) 88:1874–81. 10.1002/jmv.24544 27061822 PMC7167081

[14] Shi T Balsells E Wastnedge E Singleton R Rasmussen ZA Zar HJ. Risk factors for respiratory syncytial virus associated with acute lower respiratory infection in children under five years: systematic review and meta-analysis. J Glob Health. (2015) 5:020416. 10.7189/jogh.05.020416 26682048 PMC4676580

[15] Kuroiwa Y Nagai K Okita L Ukae S Mori T Hotsubo T. Comparison of an immunochromatography test with multiplex reverse transcription-PCR for rapid diagnosis of respiratory syncytial virus infections. J Clin Microbiol. (2004) 42:4812–4. 10.1128/JCM.42.10.4812-4814.2004 15472348 PMC522362

[16] Hirsh S Hindiyeh M Kolet L Regev L Sherbany H Yaary K. Epidemiological changes of respiratory syncytial virus (RSV) infections in Israel. PLoS ONE. (2014) 9:e90515. 10.1371/journal.pone.0090515 24594694 PMC3940902

[17] Turkish Neonatal S The seasonal variations of respiratory syncytial virus infections in Turkey: a 2-year epidemiological study. Turk J Pediatr. (2012) 54:216–22. 10.5152/TurkPediatriArs.2018.6939 23094529

[18] Hasegawa K Mansbach JM Teach SJ Fisher ES Hershey D Koh JY. Multicenter study of viral etiology and relapse in hospitalized children with bronchiolitis. Pediatr Infect Dis J. (2014) 33:809–13. 10.1097/INF.0000000000000293 24577039 PMC4145057

[19] Jones M Castile R Davis S Kisling J Filbrun D Flucke R. Forced expiratory flows and volumes in infants. Normative data and lung growth. Am J Respir Crit Care Med. (2000) 161 (2 Pt 1):353–9. 10.1164/ajrccm.161.2.9903026 10673171

[20] Wang EE Law BJ Boucher FD Stephens D Robinson JL Dobson S. Pediatric Investigators Collaborative Network on Infections in Canada (PICNIC) study of admission and management variation in patients hospitalized with respiratory syncytial viral lower respiratory tract infection. J Pediatr. (1996) 129:390–5. 10.1016/S0022-3476(96)70071-9 8804328

[21] Simoes EA. Environmental and demographic risk factors for respiratory syncytial virus lower respiratory tract disease. J Pediatr. (2003) 143 (5 Suppl.):S118–26. 10.1067/S0022-3476(03)00511-0 14615710

[22] Papenburg J Hamelin MÈ Ouhoummane N Carbonneau J Ouakki M Raymond F. Comparison of risk factors for human metapneumovirus and respiratory syncytial virus disease severity in young children. J Infect Dis. (2012) 206:178–89. 10.1093/infdis/jis333 22551815 PMC7114627

[23] Ralston SL Lieberthal AS Meissner HC Alverson BK Baley JE Gadomski AM. Clinical practice guideline: the diagnosis, management, and prevention of bronchiolitis. Pediatrics. (2014) 134:e1474–502. 10.1542/peds.2014-2742 25349312

[24] Karron RA Singleton RJ Bulkow L Parkinson A Kruse D DeSmet I. Severe respiratory syncytial virus disease in Alaska native children. RSV Alaska Study Group. J Infect Dis. (1999) 180:41–9. 10.1086/314841 10353859

[25] Carbonell X Fullarton JR Gooch KL Figueras-Aloy J. The evolution of risk factors for respiratory syncytial virus-related hospitalisation in infants born at 32-35 weeks' gestational age: time-based analysis using data from the FLIP-2 study. J Perinatal Med. (2012) 40:685–91. 10.1515/jpm-2011-0248 23093079

[26] Bradley JP Bacharier LB Bonfiglio J Schechtman KB Strunk R Storch G. Severity of respiratory syncytial virus bronchiolitis is affected by cigarette smoke exposure and atopy. Pediatrics. (2005) 115:e7–14. 10.1542/peds.2004-0059 15629968

[27] Semple MG Taylor-Robinson DC Lane S Smyth RL. Household tobacco smoke and admission weight predict severe bronchiolitis in infants independent of deprivation: prospective cohort study. PLoS ONE. (2011) 6:e22425. 10.1371/journal.pone.0022425 21811609 PMC3139660

[28] Carbonell-Estrany X Fullarton JR Gooch KL Vo PG Figueras-Aloy J Lanari M. Effects of parental and household smoking on the risk of respiratory syncytial virus (RSV) hospitalisation in late-preterm infants and the potential impact of RSV prophylaxis. J Matern Fetal Neonatal Med. (2013) 26:926–31. 10.3109/14767058.2013.765850 23379728

[29] DiFranza JR Masaquel A Barrett AM Colosia AD Mahadevia PJ. Systematic literature review assessing tobacco smoke exposure as a risk factor for serious respiratory syncytial virus disease among infants and young children. BMC Pediatr. (2012) 12:81. 10.1186/1471-2431-12-81 22721493 PMC3411420

[30] Maedel C Kainz K Frischer T Reinweber M Zacharasiewicz A. Increased severity of respiratory syncytial virus airway infection due to passive smoke exposure. Pediatr Pulmonol. (2018) 53:1299–306. 10.1002/ppul.24137 30062859 PMC6175106

[31] Foley D Best E Reid N Berry MMJ. Respiratory health inequality starts early: the impact of social determinants on the aetiology and severity of bronchiolitis in infancy. J Paediatr Child Health. (2018) 55:528–32. 10.1111/jpc.14234 30264506

[32] Lanari M Giovannini M Giuffre L Marini A Rondini G Rossi GA. Prevalence of respiratory syncytial virus infection in Italian infants hospitalized for acute lower respiratory tract infections, and association between respiratory syncytial virus infection risk factors and disease severity. Pediatr Pulmonol. (2002) 33:458–65. 10.1002/ppul.10047 12001280

[33] Sanchez-Luna M Elola FJ Fernandez-Perez C Bernal JL Lopez-Pineda A. Trends in respiratory syncytial virus bronchiolitis hospitalizations in children less than 1 year: 2004-2012. Curr Med Res Opin. (2016) 32:693–8. 10.1185/03007995.2015.1136606 26709735

[34] American Academy of Pediatrics Committee on Infectious D, American Academy of Pediatrics Bronchiolitis Guidelines C Updated guidance for palivizumab prophylaxis among infants and young children at increased risk of hospitalization for respiratory syncytial virus infection. Pediatrics. (2014) 134:415–20. 10.1542/peds.2014-1665 25070315

[35] Mauskopf J Margulis AV Samuel M Lohr KN. Respiratory syncytial virus hospitalizations in healthy preterm infants: systematic review. Pediatr Infect Dis J. (2016) 35:e229–38. 10.1097/INF.0000000000001163 27093166 PMC4927309

[36] Meissner HC Viral bronchiolitis in children. N Engl J Med. (2016) 374:1793–4. 10.1056/NEJMra1413456 27144864

[37] Gürkan F Kiral A Dagli E Karakoç F. The effect of passive smoking on the development of respiratory syncytial virus bronchiolitis. Eur J Epidemiol. (2000) 16:465–8. 10.1023/A:1007658411953 10997834

[38] Fjaerli HO Farstad T Bratlid D. Hospitalisations for respiratory syncytial virus bronchiolitis in Akershus, Norway, 1993-2000: a population-based retrospective study. BMC Pediatr. (2004) 4:25. 10.1186/1471-2431-4-25 15606912 PMC544884

